# Editorial: Drug delivery systems for mineralized tissue regeneration

**DOI:** 10.3389/fbioe.2026.1826772

**Published:** 2026-03-17

**Authors:** Qian Li, Yan Jing, Changcan Shi

**Affiliations:** 1 Chemical and Biomedical Engineering Department, University of Missouri, Columbia, MO, United States; 2 NextGen Precision Health Institute, University of Missouri, Columbia, MO, United States; 3 Department of Orthodontics, Texas A&M College of Dentistry, Dallas, TX, United States; 4 Wenzhou Institute, University of Chinese Academy of Sciences, Wenzhou, Zhejiang, China; 5 Joint Center of Translational Medicine, Wenzhou Institute, University of Chinese Academy of Sciences, Wenzhou, Zhejiang, China

**Keywords:** biomaterials, bone regeneration, drag delivery system, mineralization, tissue engineering

Mineralized tissue regeneration represents a multifaceted biological process that is essential for restoring the structure and function of skeletal and dental tissues, including bone, dentin, enamel, and cementum (Olaru et al., 2021). Although these tissues share mineralized components, their regenerative capacity varies substantially depending on tissue type, anatomical location, and pathological context. Bone regeneration, while relatively robust under physiological conditions, becomes severely impaired in large defects, inflammatory environments, infection, or systemic disease (Newman et al., 2021). Chronic inflammatory conditions such as periodontitis or metabolic disorders including diabetes can disrupt the coordinated processes of osteogenesis, angiogenesis, and immune regulation, thereby delay bone healing and compromising the quality of newly formed tissue. In contrast, dental mineralized tissues such as cementum and enamel exhibit inherently limited regenerative potential, rendering their repair particularly challenging in clinical practice. Enamel, once formed by ameloblasts during tooth development, lacks living cells in mature tissue and therefore cannot regenerate after damage. Cementum, although capable of limited repair through cementoblast activity, often fails to regenerate completely after periodontal destruction due to the complex architecture of the periodontal ligament–cementum interface and the persistent inflammatory microenvironment. Consequently, regeneration of dental mineralized tissues frequently requires advanced therapeutic strategies, including biomaterials, growth factors, stem cell–based approaches, and controlled drug delivery systems. These emerging strategies aim to recreate a favorable microenvironment that supports cell recruitment, differentiation, and matrix mineralization, thereby improving the predictability of mineralized tissue repair in both skeletal and dental applications.

Traditional therapeutic strategies—ranging from autografts and allografts to xenogeneic materials and inert implants—have primarily focused on structural replacement. However, accumulating clinical and experimental evidence indicates that successful regeneration requires more than physical filling of defects (Einhorn, 2006). The inability of conventional approaches to actively modulate inflammation, cellular recruitment, angiogenesis, and matrix mineralization often results in delayed healing, compromised integration, or incomplete functional restoration. These limitations have driven increasing interest in biologically instructive strategies capable of regulating the local regenerative microenvironment. Drug delivery systems (DDS) have therefore emerged as a central paradigm in mineralized tissue regeneration (Anil et al., 2015). By enabling localized, sustained, and spatially controlled delivery of bioactive molecules, DDS offer a means to precisely influence key biological events such as immune modulation, stem or progenitor cell behavior, and lineage-specific differentiation. Importantly, the effectiveness of many regenerative cues depends not only on their dosage but also on their temporal presentation and interaction with the surrounding tissue environment. This recognition has shifted the focus of regenerative medicine from passive scaffolding toward dynamic, responsive delivery platforms.

Recent advances in biomaterials and nanotechnology have substantially expanded the design landscape of DDS for mineralized tissues. Contributions within this Research Topic exemplify this progress across multiple material scales and application contexts. Scaffold-based delivery systems, including electrospun and composite architectures, demonstrate how structural matrices can be functionalized with osteoinductive or bioactive agents to support bone regeneration while reducing reliance on traditional barrier membranes or particulate grafts (Slavin et al.). In parallel, systematic analyses of 3D-printed scaffolds highlight how precise control over mechanical properties, porosity, and material composition can be leveraged to meet both biological and delivery requirements for complex bone defects (Wang et al.).

At the nanoscale, delivery platforms such as carbon dots and supramolecular peptide assemblies illustrate the growing trend toward multifunctional systems. Carbon dots-based DDS integrate drug delivery, osteogenic stimulation, and bioimaging capabilities, underscoring their potential as versatile nanocarriers for bone regeneration (Liu et al.). Supramolecular peptide nanofiber hydrogels further demonstrate how molecular self-assembly can be exploited to mimic extracellular matrix features while incorporating bioactive motifs that regulate osteogenesis, angiogenesis, and immune responses (Wan et al.).

Beyond bone regeneration, this Research Topic deliberately extends DDS concepts to other mineralized tissues within the craniofacial complex. Morphological investigations into cementum development provide fundamental insights into epithelial–mesenchymal interactions and periodontal attachment, offering a biological framework for future delivery-based strategies in periodontal regeneration (Zhan et al.). In a preventive and translational context, sustained-release systems designed for enamel protection—such as fluoride-releasing orthodontic devices—demonstrate how controlled delivery principles can be applied to address mineral loss during clinical interventions (Strickland et al.).

Importantly, the Research Topic also captures clinically oriented delivery strategies, as exemplified by local antibiotic-loaded bone substitutes used to control infection while simultaneously promoting bone regeneration. Such studies highlight the translational relevance of DDS in complex clinical scenarios where infection control and tissue repair must be addressed concurrently (Su et al.).

Collectively, these contributions reflect the evolving role of drug delivery systems as active regulators of mineralized tissue regeneration. Rather than serving as isolated solutions, the studies within this Research Topic form a cohesive narrative that spans material innovation, biological understanding, and clinical translation. Together, they emphasize that future advances in mineralized tissue regeneration will depend on delivery systems that are not only structurally supportive, but also biologically responsive, tissue-specific, and adaptable to diverse regenerative challenges.
